# Hodgkin's lymphoma masquerading as vertebral osteomyelitis in a man with diabetes: a case report

**DOI:** 10.1186/1752-1947-4-102

**Published:** 2010-04-06

**Authors:** Rachel A Bender Ignacio, Anne Y Liu, Aliyah R Sohani, Jatin M Vyas

**Affiliations:** 1 Massachusetts General Hospital, Department of Medicine, Gray Building Room 740, 55 Fruit Street, Boston, MA 02114, USA; 2 Massachusetts General Hospital, Division of Infectious Disease, Grey-Jackson Room 504, 55 Fruit Street, Boston, MA 02114, USA; 3 Massachusetts General Hospital, Department of Pathology, Gray-Jackson Room 148-B, 55 Fruit Street, Boston, MA 02144, USA

## Abstract

**Introduction:**

Infection and malignancy often have common characteristics which render the differential diagnosis for a prolonged fever difficult. Imaging and tissue biopsy are crucial in making a correct diagnosis, though differentiating between chronic osteomyelitis and malignancy is not always straightforward as they possess many overlapping features.

**Case Presentation:**

A 52-year-old Caucasian man was treated with antibiotics for his diabetic foot infection after a superficial culture showed *Staphylococcus aureus*. He had persistent fevers for several weeks and later developed acute onset of back pain which was treated with several courses of antibiotics. Radiographic and pathological findings were atypical, and a diagnosis of Hodgkin's lymphoma was made 12 weeks later.

**Conclusion:**

Clinicians should maintain a suspicion for Hodgkin's lymphoma or other occult malignancy when features of presumed osteomyelitis are atypical. Chronic vertebral osteomyelitis in particular often lacks features common to acute infectious disease processes, and the chronic lymphocytic infiltrates seen on histopathology have very similar features to Hodgkin's lymphoma, highlighting a similar inflammatory microenvironment sustained by both processes.

## Introduction

Osteomyelitis of the spine is caused by direct instrumentation to the area, or contact with overlying soft-tissue infection, or by hematogenous seeding of the vertebrae. Risk factors for hematogenous vertebral osteomyelitis (HVO) include prolonged bacteremia, indwelling catheters, underlying diabetes, malignancy, or other immunocompromised states [1]. Several other disease processes can also present with vertebral lesions, including atypical infections and primary or metastatic malignancy. Hodgkin's lymphoma (HL) can present with asymptomatic mass lesions, B-symptoms or local symptoms in the location of the tumor bulk. It is most prevalent in young to middle-aged men. Lymphoma is not commonly found in the bone at presentation, and B-cell non-HLs are much more likely than HL to present in the bone. There is scant literature directly addressing bony lesions in HL, especially in comparison to infectious disease processes [2]. This case demonstrates a patient who had the classic presentation and risk factors for HVO, but was ultimately found to have HL. The diagnostic difficulties, histology of biopsy samples, radiographic findings and disease similarities are discussed.

## Case Presentation

A 52-year-old Caucasian man presented at an outside hospital with three days of fevers and a swollen, purpuric right foot. He had noted a necrotic-appearing ulcer on the plantar surface of his fifth digit one week previously. His past history was remarkable for diabetes mellitus type 2 (his last hemoglobin A1c [HbA1c] test was 7.0%), his right great toe had been amputated secondary to infection in 2001; and he had a previous cigarette use of 60 pack-years. He had worked for the United States Forest Service, doing physical labor, often working in wet boots and with close contact to the feces of several species of forest animals. He had suffered a tick bite 6 months previously.

On admission he was found to have a leukocytosis of 19,000 cells/mm^3 ^and mild normocytic anemia. The foot ulcer was superficially cultured and grew methicillin-sensitive *Staphylococcus aureus *(MSSA). While hospitalized, he experienced chills, night sweats, nausea and vomiting. He was discharged and given cefazolin and metronidazole for a planned six-week course following normalization of his leukocyte count and resolution of systemic symptoms.

One month after discharge, he was re-admitted for evaluation of recurrent sweats, chills and weakness. His peripherally inserted central catheter (PICC) was removed, and the tip was cultured but yielded no growth. Surgical debridement of the fifth digit revealed no gross purulence, and broth from the deep tissue culture grew only *Bacillus *species. Vancomycin and piperacillin-tazobactam were substituted for the cefazolin and metronidazole regimen for a planned six weeks duration of therapy.

**Figure 1:**
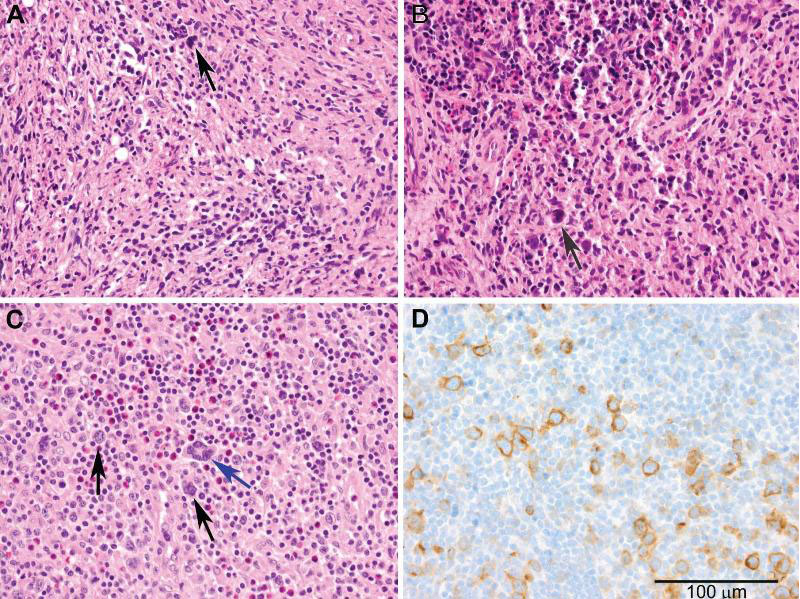
**Representative tissue samples at 400× magnification**. The initial L4 vertebral core biopsy (a) shows marrow replacement by a mixed inflammatory infiltrate consisting of small lymphocytes and some neutrophils in a fibrotic background. Rare large cells are present (arrow), but diagnostic Reed-Sternberg (RS) cells are not identified. Trilineage hematopoietic marrow was present in other areas (not shown). The right inguinal lymph node core biopsy (b) demonstrates a mixed inflammatory infiltrate consisting of small lymphocytes, histiocytes and eosinophils in a fibrotic stroma. Rare large degenerated cells are also present (arrow) but are non-specific findings. The left anterior cervical lymph node excisional biopsy (c) shows architectural effacement by a polymorphous infiltrate that includes scattered eosinophils, as well as diagnostic multinucleated RS cells (blue arrow) and mononuclear variants (black arrows) that stain positively for CD30 by immunohistochemistry (d).

One week after admission, he had a new onset of lower back pain, prompting computed tomography (CT) and MRI scans of the spine which revealed diffuse bony lesions from T12 to L4. Microscopic analysis of a needle biopsy of the L4 lesion showed a mixed inflammatory infiltrate in a fibrotic background, interpreted as partially treated osteomyelitis (Figure 1a). Core needle biopsy of a right inguinal node demonstrated a dense mixed inflammatory infiltrate with rare large degenerated cells of uncertain significance (Figure 1b). Gram stain, acid-fast stain, and bacterial, mycobacterial and fungal cultures were negative from both biopsies. Antibiotics were discontinued and he was discharged.

He was re-admitted one week later with recurrent systemic symptoms and a total leukocyte count of 20,000 cells/mm^3^. Vancomycin and imipenem-cilastatin were started. Transesophageal echocardiogram revealed no valvular vegetations. A bone scan revealed multiple abnormal areas of uptake including the right foot, several ribs, scapula and both femurs. Blood cultures throughout these multiple hospitalizations did not recover any pathogenic organisms.

Upon transfer to our facility, he noted a 7 kg weight loss since the onset of symptoms, and he was fatigued but ambulatory. Examination was significant for a single <2 cm soft, mobile, tender lymph node in the left anterior cervical chain and symmetric mild lymphadenopathy in both axillae and the groin. His right foot ulcer was well healed, though mild purpura and swelling remained over the third through fifth digits. His spine was not tender to palpation. Laboratory testing revealed a leukocyte count of 18,400 cells/mm^3 ^with 85% neutrophils and a platelet count of 404,000/mm^3^. C-reactive protein (CRP) was 83 mg/L, erythrocyte sedimentation rate (ESR) 106 mm/hour, and alkaline phosphatase 461 U/L. Laboratory evaluations for tick-borne and endemic fungal infections were all negative, as were an anti-nuclear antibody (ANA) test, a rapid plasma regain (RPR) test, and a human immunodeficiency virus enzyme-linked immunosorbent assay (HIV ELISA). Intradermal purified protein derivative (PPD) did not elicit any induration. All antibiotics were discontinued, and the patient remained febrile at 38.3-39.3°C nightly with drenching sweats.

**Figure 2:**
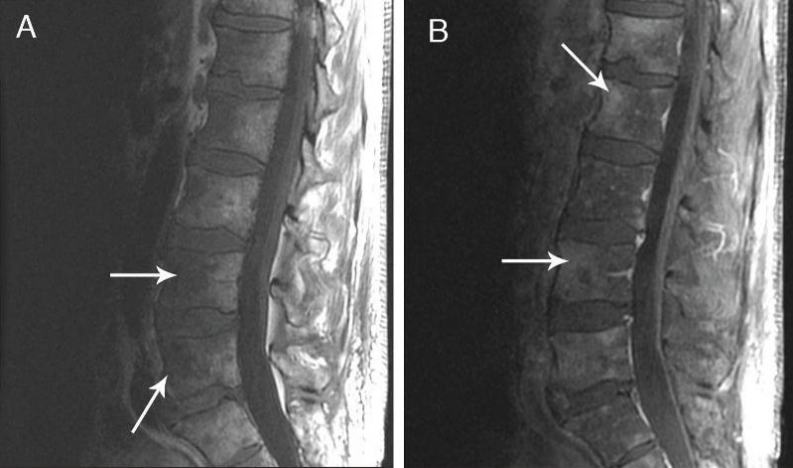
**MRI of spine demonstrating multifocal hypointensities (arrows) sparing the intervertebral disks in T1-weighted images (a)**. The same lesions (arrows) appear hyperintense on T2-weighted images (b). No inflammation of the paraspinal muscles or abscess was identified.

**Figure 3:**
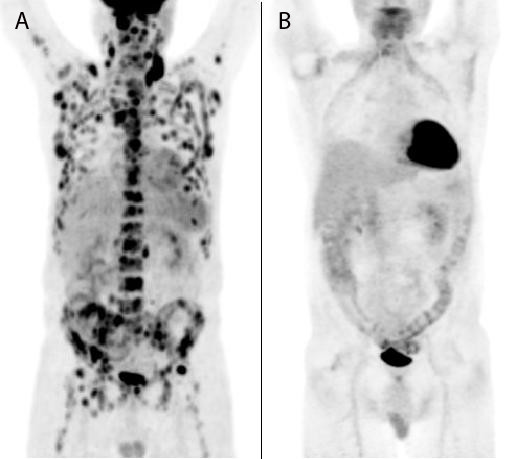
**The scout film of the positron emission tomography-computed tomography (PET-CT) scan performed prior to this first round of chemotherapy (a) demonstrates diffuse regions of uptake involving multiple ribs, multiple vertebral bodies, the pelvis, the sternum and the scapula**. There is also increased fluorodeoxyglucose (FDG) uptake in multiple bilateral lymph node regions extending from the jugular, supraclavicular, mediastinal, retroperitoneal, pelvic and inguinal regions, consistent with Hodgkin's lymphoma. There is increased FDG uptake in the posterior and right lateral walls of the nasopharynx. About two months after his first round of chemotherapy, a repeat PET-CT scan (b) showed a marked interval decrease in the FDG-avid metastatic burden.

Ten sets of blood cultures were negative for bacteria, fungi and mycobacteria. CT scans of the chest, abdomen and pelvis revealed multiple small pulmonary nodules, bilateral small pleural effusions, a small pericardial effusion, two small calcified granulomas in the liver, and diffuse cervical, mediastinal, iliac and inguinal adenopathy (all ≤1.6 cm). A repeat MRI of the spine confirmed multiple areas of T1 hypointensity and T2 enhancement throughout the cervical, thoracic and lumbar spine, sparing the intervertebral disks and the cord (Figure 2). Microscopic examination of a left cervical lymph node excisional biopsy and staging posterior iliac crest bone marrow biopsy revealed the presence of large atypical cells consistent with Reed-Sternberg (RS) cells and variants, and a diagnosis of Stage IV mixed-cellularity classical HL was made (Figures 1c and 1d). Positron emission tomography (PET)-CT was performed, displaying innumerable lesions in the axial spine and fluorodeoxyglucose (FDG)-avid nodes throughout innumerable lymphatic chains. Increased uptake was especially noted in the right lateral nasopharynx, without other solid organ involvement. An escalated BE(A)COPP (bleomycin, etoposide, doxorubicin, cyclophosphamide, vincristine, procarbazine, and prednisone) regimen was initiated. Because of hematologic complications, our patient completed a course of modified Adriamycin [doxorubicin], bleomycin, vinblastine and dacarbazine (ABVD) chemotherapy and is in clinical and radiographic remission (Figure 3).

## Discussion

Though it is common for malignancies and systemic infections to have overlapping features, several aspects of our case proved to be unique, ultimately delaying the diagnosis of HL in our patient. A progression from what became a chronic diabetic foot infection to vertebral osteomyelitis would have been logical. His underlying diabetes, partially treated infection, and ultimately discovered malignancy likewise placed him at significant risk for hematogenously seeded vertebral osteomyelitis [1]. Additionally, our patient denied 'B symptoms' prior to the appearance of his ulcer, and his leukocytosis and fevers temporarily resolved with initiation of each new antibiotic regimen, making the diagnosis of malignancy less likely. The presence of *S. aureus *in his necrotic diabetic foot ulcer concurrent with fevers then directed treatment of subsequent fevers exclusively towards persistent bacterial infection for several weeks.

Patients diagnosed with non-HIV associated HL are also diagnosed with twice as many infections in the 10 years prior to diagnosis as age-matched counterparts without malignancy, not including the year preceding diagnosis [3]. It is interesting to note that herpesviridae infections are more prominent in this population, presumably as a result of subtle immunological defects from their malignancy.

*S. aureus *accounts for nearly half of all cases of HVO and most commonly presents with back pain (89%) and fever (>60%) [4]. Initial characteristics of CT and MRI scans in our case raised suspicion for systemic involvement, though both a vertebral fine needle aspirate and core biopsy failed to confirm a diagnosis. Radiographic features of osseous HL and HVO are often indistinguishable [2,5]. Evidence of spondylodiscitis, though classic for infection, is not uniformly present in HVO, and is often absent without involvement of contiguous vertebrae. Imaging demonstrating paraspinal inflammation increases sensitivity and specificity for HVO, but atypical organisms, such as mycobacteria, often lack paraspinal inflammation and are also more likely to demonstrate multi-level disease and skip lesions [6].

In HL, the most common site of bony involvement is the spine, and multiple lesions at presentation are more common than a solitary lesion [7]. Radiographic features of our case made malignancy more likely, yet bone involvement at presentation of HL is quite uncommon, with only 33 cases being identified by biopsy in the last 70 years at the Mayo Clinic, with the majority of cases being primary osseous HL. When HL presented simultaneously in an osseous and a non-osseous site, 25% of such cases were initially misdiagnosed as osteomyelitis by histopathology [2]. Fine needle aspirates revealing lymphoma cells have a nearly perfect diagnostic accuracy, while those containing non-specific findings of osteomyelitis have insufficient positive or negative predictive value to confirm or exclude malignancy [8]. The infrequently encountered, often degenerated, malignant cells in our patient's initial biopsies illustrate the need for a high index of suspicion in such cases, and the importance of procuring additional tissue via excisional biopsy to confirm a diagnosis. The only site of solid organ involvement in our case proved to be within the nasopharynx, which is found to be the site of the primary lesion in less than 1% of all HL cases.

The diagnostic difficulties above highlight the similar molecular pathways of chronic inflammations seen in osteomyelitis and in HL. The microenvironment of HL is composed of a heterogeneous group of cells including T cells (CD4+ T cells being the most prominent cell type), B cells, plasma cells, neutrophils, eosinophils and mast cells [9]. The prototypical RS cell represents only about 1% of the cells in the HL tumor. The expression of multiple cytokines by the RS cells appears to be critical in the development of the microenvironment and these other cell types appear to be required to sustain the viability of the RS cells [10]. It is interesting to note that RS cells survive in immunocompetent, but not immunodeficient, mice. RS cells secrete interleukin-8 (IL-8) which serves as a chemoattractant for neutrophils, and express multiple chemokine ligands including CCL5, CCL17 and CCL22, which attract certain T-cell subsets [9]. Osteomyelitis is more frequent in persons carrying the particular polymorphism of the Bax gene promoter also linked to the failure of these malignant cells to undergo apoptosis [11]. Increased serum levels of IL-6 found in patients with active osteomyelitis play a causative role in decreased peripheral blood neutrophil apoptosis [12]. Both diseases produce a self-sustaining microenvironment that is reliant on competent host immunity to produce long-lived inflammatory cells generated by altered cell signaling.

## Conclusion

Our case of Stage IV HL masquerading as osteomyelitis highlights the inherent difficulties in differentiating bone infection from malignant infiltration. Histopathological confirmation of HL only came after an inconclusive spinal biopsy and a lymph node core biopsy showing only rare atypical cells. Clinicians should maintain a suspicion for HL or other occult malignancy in patients with presumed osteomyelitis whose bony lesions appear atypical when analyzed by radiography or pathology, or in their response to treatment.

## Consent

Written informed consent was obtained from the patient for publication of this case report and any accompanying images. A copy of the written consent is available for review by the Editor-in-Chief of this journal.

## Competing interests

The authors declare that they have no competing interests.

## Authors' contributions

RABI researched the topic, organized the paper, and prepared the radiographic images. AYL, RABI and JMV cared for the patient during his hospital admission. RABI and ARS prepared the histological samples for publication. All authors read and reviewed the final manuscript.
